# The association between home modifications and depression among older people in China

**DOI:** 10.3389/fpubh.2025.1475037

**Published:** 2025-05-15

**Authors:** Xuanru Lyu, Jian Sun

**Affiliations:** ^1^School of Public Administration, Jilin University, Changchun, Jilin, China; ^2^Law School, Ningbo University, Ningbo, Zhejiang, China; ^3^Donghai Academy, Ningbo University, Ningbo, Zhejiang, China

**Keywords:** home modifications, depression, older people in China, propensity score matching, China Longitudinal Aging Social Survey

## Abstract

**Background:**

As individuals age, their living environments often become inadequate to meet the evolving demands associated with aging. An accommodating home environment is crucial for ensuring the health and well-being of older people. Home modifications effectively create a supportive living space for those who choose to age in place. The objective of the study is to examine the association between home modifications and the depression levels of older people in China.

**Methods:**

Using data from 5,706 urban older people collected in the 2018 China Longitudinal Aging Social Survey (CLASS), this study employs a multivariate ordered logistic regression model and propensity score matching (PSM) to assess the association between home modifications and depression levels among older people in China.

**Results:**

The results suggest that home modifications are significantly associated with a reduction in depression among older people, with a greater degree of modification correlating with more substantial reductions in depressive symptoms. These findings remained consistent across various models and robustness checks, suggesting that modifying the living environment can significantly benefit older people’ mental health.

**Conclusion:**

These findings demonstrate the significance of the implementation of home modifications as a practical intervention for improving mental health by reducing depression levels among older people in China.

## 1 Introduction

Population aging is one of the most significant demographic trends of the 21st century. China is rapidly graying, which has been an essential and irreversible social development trend for a long time. By the end of 2023, the population of individuals aged 60 and above in China had surpassed 296 million, representing 21.1 percent of the total population. China faces urgent challenges due to its large older population and rapidly aging population. The demographic shift raises significant concerns regarding the provision of supportive social services for older people (1). Among these challenges, the concept of “aging in place,” or enabling older people to remain in their homes as they age, has emerged as a central strategy for care for older people in China. The Landscape of Older People Care Service Provision in China shows that 90% of older people prefer to live in their own homes during retirement, around 7% depend on community support, and 3% receive care in institutions, which indicates that more older people will age at home, which is cost-effective and enhances their control and identity over daily life, ultimately positively affecting their well-being and mental health (2, 3).

Globally, aging in place has gained recognition as a key strategy for older people care, as emphasized by the Madrid International Plan of Action on Ageing (4). The center of aging in place is the role of the home environment. The home environment is widely recognized as a primary factor that impacts human health (5). Both policymakers and researchers have broadly acknowledged the critical role of age-friendly housing in enhancing the well-being of older people and adapting homes to meet older people’ needs (6).

As the Chinese population ages, the demand for housing that provides a safe and comfortable living environment for older people is expected to rise significantly. Home modifications such as sufficient wide hallways to accommodate wheelchairs, first-floor bedrooms to minimize the need for staircase navigation, and bathrooms equipped with railings or seats to prevent falls are critical for safety (7). However, as individuals age, the likelihood of falls increases, particularly within their homes (8). For instance, studies in the United States show that approximately 30% of adults aged 65 and older experience falls annually, with the proportion rising to 50% among those aged 80 and above (9). In South Korea, one of the fastest-aging populations globally (9), 60.5% of falls among adults aged 65 and older occur within their homes (10). In China, around 40 million falls are estimated to occur annually among older people in urban areas, primarily within their own homes, and a considerable proportion of these falls are linked to hazardous conditions in residences over 20 years old (11). Falls among older people often necessitate long-term recovery and, in some cases, can result in fatal outcomes (12). Unsafe living environments significantly increase the risk of falls and heighten the fear of falling, particularly among individuals who previously experienced such incidents (13). This fear often leads to a decline in physical activity and mobility, eroding confidence and self-efficacy and ultimately contributing to depression and other adverse mental health outcomes (14, 15). These challenges not only undermine the mental health of older people but also impede the ability of older people to live independently, obstructing successful aging in place (16). Despite the growing demand for safer and more accessible living environments, most Chinese homes remain inadequately equipped to meet the needs of aging populations, with critical safety and accessibility features often lacking (17).

There is a growing global recognition of the importance of physical and social environments in supporting the well-being of older individuals (18, 55). In 2007, the World Health Organization (WHO) underscored the significance of constructing inclusive environments for individuals aged 65 and above to facilitate their ability to carry out all essential life activities and achieve their utmost capabilities (19). Indeed, the home serves as the central point of older people’ lives, offering a sense of control, individuality, personhood, and security for their functional, mental, and subjective well-being (20–22). Prior research has extensively examined the significance of the domestic setting for older people (23–25). Numerous research has shown that home modifications can address various hazards that contribute to falls, such as slippery floors and narrow doorways, thereby enhancing the overall safety and independence of older people (24, 26–28). Beyond providing physical conception of space like shelter; home also has an unignorable impact on numerous mental health outcomes (29). Bonnefoy (5) found that living conditions and housing environment serve as the foundation for numerous aspects that impact residents’ health. While the association between home modifications and physical health has been extensively studied, their association with mental health, particularly in the Chinese context, remain underexplored.

Local governments in China have taken the lead in implementing aging-friendly home modifications. For example, in 2016, Beijing released the Beijing Home-Based Elderly Care Service Regulations, which explicitly mandated aging-friendly renovations in residential buildings, including improvements to accessibility in older communities and providing government-funded home modifications for economically disadvantaged older adults. Similarly, in 2018, the municipal governments of Chengdu and Shanghai launched citywide home modification initiatives to enhance home accessibility. At the national level, in 2020, nine Ministries jointly issued the Guiding Opinions on Accelerating the Implementation of Home-Based Aging-Friendly Renovation Projects, emphasizing the urgency of advancing aging-friendly home modifications and providing a recommended list of modification projects and age-friendly products. More recently, in 2024, the Ministry of Civil Affairs of the People’s Republic of China released the General Requirements for Elderly-Oriented Renovation of Elderly’s Home Environment’ (hereafter referred to as ‘The Requirements’). According to these policies, home modifications (HM) are defined as activities designed to improve the living environment for older people through alterations to physical space, the installation of appropriate facilities and equipment, and the provision of age-friendly products. Home modifications effectively establish a supportive living environment, enabling older people to remain in their homes by minimizing challenges and eliminating barriers (30). Moreover, the home environment significantly influences an older adult’s ability to live independently and maintain physical functions (31). Previous studies have defined home modifications as alterations to living spaces designed to improve usability, safety, and independence for older people (32, 33). Guided by the Requirements, home modifications include specific measures such as eliminating height differences between the bedroom and living room, providing adequate space for wheelchair turning at room junctions, ensuring sufficient lighting in areas with wall corners, height differences, or potential slip hazards, and installing grab bars based on mobility needs and family conditions. The scope of HM encompasses several major categories, including floors, stairs and hallways, doors and windows, lighting, electrical outlets and switches, safety handrails, entryways, living rooms, kitchens, dining areas, and balconies. The primary objective of HM is to create a supportive living environment that enhances safety and accessibility for older people. These modifications specifically focus on preventing falls by installing grab bars and eliminating tripping hazards, improving accessibility with features such as ramps and wider doorways, and promoting independence through incorporating lever-style door handles and handrails.

Despite the well-documented benefits of home modifications, there has been limited research into their specific association with the mental health of older people, particularly within the context of China. Some studies suggest that the implementation of home modifications for older people faces challenges. For instance, Kruse et al. (34) found that many older Americans are reluctant to change their homes, often preferring to rely on their ideas rather than seeking advice from home remodeling programs. Successful implementation and acceptance of home modifications by older people depend on accurately identifying their specific needs, as they have valuable expertise and personal insight into policies and care services based on their life experience (35). Given the significant role of mental health in the overall well-being of older people, it is essential to explore the broader benefits of home modifications, particularly their potential association with the reduction of depressive symptoms among older people.

This study aims to address this gap by empirically investigating the association between home modifications and the mental health of older people in China, utilizing data from the 2018 China Longitudinal Aging Social Survey (CLASS). The study makes three primary contributions. First, it explores the relatively underexamined area of how home modifications are associated with the mental health of older people. Second, it evaluates the association between home modifications and depression levels and investigates how varying degrees of modification are associated with these levels. Finally, it employs the propensity score matching (PSM) model to correct for potential estimation biases arising from participant self-selection in the study.

## 2 Method

### 2.1 Data source and sample selection

**Figure 1:**
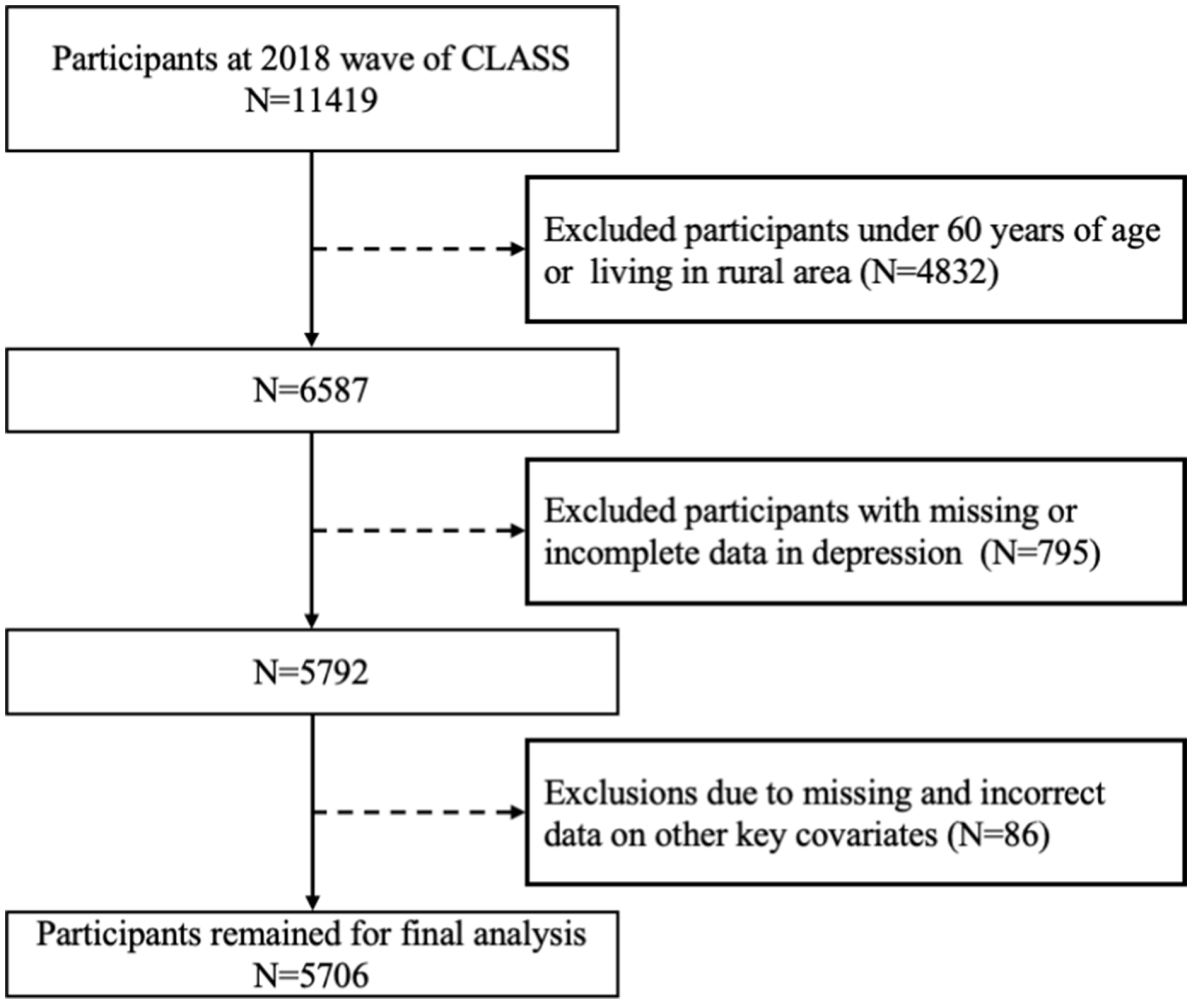
Sampling flow chart.

This research uses data acquired from the 2018 China Longitudinal Aging Social Survey (CLASS), which was executed by the Renmin University of China. The survey focuses on Chinese individuals aged 60 and above. The socioeconomic background information of older Chinese individuals is systematically collected using a stratified multistage probability sampling technique. Our study selects older people who have implemented home modifications as the research participants based on the research objectives and requirements. After excluding samples with data that was missing, unwillingness to respond, and evident mistakes in crucial variables, this study’s final analysis sample size was 5,706. Figure 1 depicts a flow chart that illustrates the process of selecting the research population.

### 2.2 Measures

#### 2.2.1 Independent variables

##### 2.2.1.1 Home modification

This study uses home modifications as an independent variable. The CLASS questionnaire includes the following questions: “Is there a wheelchair-accessible ramp at the entrance of your building’s hallway?” “Does your current residence have issues with thresholds or uneven flooring?” “Is there a handrail installed in the house where you currently reside? (e.g., in the toilet, bathroom, or hallways at home)” “Is the toilet you currently use a flush toilet?” “Does your current residence have an emergency call device?” Responses for the first four questions were coded: “Yes” = 1, “No” = 0. The fifth question was reverse-coded: “Yes” was assigned a value of 0, and “No” was assigned a value of 1. The total score for home modifications was calculated by summing the values from these five items. A score of 1 or higher indicates that the residence has undergone modifications and is assigned a value of 1. In contrast, a total score of 0 suggests that the older adult’s residence has not undergone any modifications and is assigned a value of 0.

##### 2.2.1.2 Degree of home modification

The degree of home modification is quantified based on the total score derived from the five questions mentioned above, with the total score ranging from 0 to 5. A higher total score reflects a greater degree of home modification.

#### 2.2.2 Dependent variable depression

The primary dependent variable in this research was depression levels among older people. The Center of Epidemiological Survey-Depression Scale (CES-D) is widely used to evaluate depressive symptoms in the general population (36, 37). The measure has exhibited robust internal consistency across many populations and demonstrated simultaneous reliability in developing and developed nations (38). During the CLASS study, participants were required to respond to a twelve-question questionnaire, including three items about positive emotions and nine concerning depression. The scale consisted of four response options for each item: “None of the time,” “Some or a little of the time,” “Occasionally or a moderate amount of time,” and “Unable to answer.” In this study, we excluded samples in which respondents selected “unable to answer.” For the depression item, we assigned integer values between 1 and 3 to the remaining responses, and for the positive item, we carried out a corresponding reverse assignment. Consequently, the CES-D scores ranged from 9 to 27, the scores higher indicating a higher degree of depression.

#### 2.2.3 Covariates

Following previous studies (39–42), the control variables of this study are selected from four dimensions: demographic characteristics, socioeconomic status, physical health, and family structure. Demographic characteristics include age, gender, marital status, ethnicity, and education. Gender was coded as 1 for male and 0 for female. Marital status was coded as 1 for individuals who have a spouse and are married, and 0 for others (widowed, divorced, or unmarried). Education was coded as: Illiterate = 0, elementary school /old-style private school = 1, junior high school = 2, high school or higher = 3. Ethnicity was coded as 1 for Han nationality and 0 for non-Han nationality. Socioeconomic status is measured by pension income monthly (43), which includes various social security benefits, such as Basic Pension Insurance for Urban Enterprise Employees, Pension Insurance for Employees of Government and Public Institutions, Basic Pension Insurance for Urban and Rural Residents. A logarithmic transformation of pension income was applied. Physical health is measured using Activities of Daily Living (ADL) (44) and hospitalization (45). ADL is coded as 1 for individuals with limitations and 0 for those without limitations. Hospitalization is coded as 1 for individuals hospitalized in the past 2 years and 0 otherwise.

Additionally, the study considered the family structure (46). Steptoe et al. (47) found that loneliness and social isolation are associated with an increased mortality risk among older people. Families composed solely of older people may experience reduced social networks, increasing the likelihood of loneliness and social isolation. Family structure is represented by “living situation,” coded as 1 for couples living together or a single older adult living alone and 0 for other arrangements. The number of surviving children is also included, as it may affect mental health by alleviating loneliness and enhancing life meaning (48) or potentially increasing economic hardship and physical pain (49, 50).

Table 1 displays the assignment and descriptive analysis of variables.

**Table 1:** Variable definitions and descriptive statistics.

Variables	Definitions	Mean			Mean-diff	*p* value
		Total	Beneficiaries	Non-beneficiaries		
		(*N* = 5,706)	(*N* = 3,871)	(*N* = 1835)		
Depression	Score from 9 to 27	15.38	15.019	16.142	1.123	0.000***
HM	Adopt at least one of the home modification items = 1	0.678	1	0	−1	.
HM degree	Score from 0 to 5	1.065	1.57	0	−1.57	0.000***
Age	Unit; years	71.185	71.332	70.874	−0.458	0.028**
Gender	Male = 1	0.48	0.476	0.488	0.013	0.37
Married	Has spouse and married = 1	0.716	0.725	0.696	−0.029	0.024**
Ethnicity	The Han nationality = 1	0.968	0.976	0.951	−0.024	0.000***
Education	Illiterate = 0, elementary school /old-style private school = 1, junior high school = 2, high school and above = 3	3.242	3.412	2.884	−0.528	0.000***
Pension	Amount of pension income monthly	6323.417	7015.483	4863.478	−2,200	0.000***
ADL	ADL with limitations = 1	0.076	0.091	0.043	−0.048	0.000***
Hospitalization	Have been hospitalized in the past 2 years = 1	0.271	0.274	0.263	−0.011	0.388
Living situation	A couple living together or a single older adult living alone = 1	0.531	0.556	0.477	−0.079	0.000***
Offspring	Number of surviving children	2.261	2.137	2.523	0.386	0.000***

*p < 0.10, **p < 0.05, ***p < 0.01.

### 2.3 Statistical methods

This study investigates the impact of HM on the severity of depression among older people. The investigation was conducted in two phases. The initial phase used an multivariate ordered logistic model to examine the association between HM, the degree of HM, and depression levels among older people. The study categorized depression levels into a range of 9 to 27. The dependent variable at this time was an ordinal variable representing the severity of depression, and the logistic model was employed to evaluate the factors that influence depression in older people. The setup of the model was as follows:


(1) $$Y_{i}=\beta_{0}+\beta_{1}X_{1}+\beta_{2i}X_{2}+....+\beta_{10}X_{10}+\beta_{11}{\mathrm{HM}}_{i}+\mu_{i}$$

(2) $$Y_{i}=\beta_{0}+\beta_{1}X_{1}+\beta_{2i}X_{2}+....+\beta_{10}X_{10}+\beta_{12}\mathrm{HM}\_{\text{degree}}_{i}+\mu_{i}$$

In Equations 1 and 2, *i* represents individual observations in the sample. $Y_{i}$ represents the depression level of older people, *X*_1_, *X*_2_, …, *X*_10_ are the control variables, HM is a binary variable indicating whether an older people has adopted home modifications, and HM_degree represents the degree of home modifications, *β* denotes the coefficient vector, and *μ* represents the random error. Robust standard errors were employed to account for potential heteroscedasticity. All models were implemented using Stata 17.0.

Following the initial regression analysis, propensity score matching (PSM) was employed to assess the association between home modifications and depression levels. As introduced by Rosenbaum and Rubin (51), PSM is a counterfactual method that facilitates the evaluation of net effects between treatment and control groups. This technique is particularly useful for addressing potential biases related to model specification and self-selection. Unlike traditional multiple regression models, the PSM method is not influenced by a particular functional form, thereby reducing model-related bias. The decision to implement home modifications is typically influenced by factors such as an older people’s ADL, pension income, and demographic characteristics. In this study, the treatment group comprises older people who have adopted home modifications, while the control group includes those who have not made any changes to their homes. PSM enables us to estimate the association between home modifications and depression levels by matching treatment and control groups based on observable variables. It constructs a hypothetical scenario in which the control group closely resembles the treatment group’s situation had they not received the intervention. The primary focus is on the differences in depression levels between these groups, which represent the key outcomes of interest.

To carry out the PSM strategy, this study first estimates the propensity scores of the data assigned to the treatment group. We utilize various matching techniques to pair treatment and control groups based on these scores. There is currently no definitive set of criteria for measuring the accuracy and efficiency of different matching methods. Typically, previous studies employ various matching approaches to assess the reliability of the anticipated outcomes. A balance test was conducted to evaluate the degree of bias before and following the matching procedure, which helps assess the matching quality. High-quality matching ensures that the initial features of the treatment group and control groups are closely aligned post-matching. This alignment allows for an accurate estimation of the average treatment effect on the treated (ATT). The ATT can be approximated using the following method:


(3) $$\begin{array}{l} \mathrm{ATT}=E_{p(x_{i})|HM_{i=1}}\\ \left\{E[{\text{depress}}_{i}(1)|{\mathrm{HM}}_{i}=1,P(X_{i})]-E[{\text{depress}}_{i}(0)|{\mathrm{HM}}_{i}=0,P(X_{i})]\right\} \end{array}$$

In Equation 3, ATT represents the average treatment effects of HM on the depression of older people. ${\mathrm{HM}}_{i}$ is a binary variable, indicating whether the older adult *i* adopted HM (1 is the treatment group, 0 is the control group), ${\text{depress}}_{i}(0)$ is the depressive symptoms of the control group, ${\text{depress}}_{i}(1)$ is the depressive symptoms of the treatment group, and $X_{i}$ are covariates.

## 3 Results

### 3.1 Results of baseline regression

This study included four regression models to investigate the impact of home modifications on the depression levels of older people. In Table 2, model (a) and model (c) indicate that HM and the degree of HM are significantly associated with the depression level of older people, with a 1% level of statistical significance, suggesting that HM is related to a reduction in the depression level of older people. In model (a), without considering control variables, HM was associated with a reduction of 0.627 units in depressive symptoms among older people, significant at the 1% level. After accounting for the control variables, including demographic, physical health, socioeconomic, and family factors, the effect decreases slightly to 0.588 units, still remaining significant at the 1% level. Model (c) shows that the degree of HM is associated with a reduction of 0.255 units in depression levels (significant at the 1% level) when no control variables are included. This association increases slightly to −0.260 when control variables are incorporated in Model (d). The findings suggest that modifying the home environment is associated with a reduction in the depression level of older people.

**Table 2:** Empirical analysis of the association between home modifications and depression levels in older people.

	Model (a)	Model (b)	Model (c)	Model (d)
HM	−0.627^***^ (0.049)	−0.588^***^ (0.052)		
Degree of HM			−0.255^***^ (0.024)	−0.260^***^ (0.025)
Age		0.016^***^ (0.004)		0.014^***^ (0.004)
Gender		−0.042 (0.048)		−0.034 (0.048)
Married		−0.291^***^ (0.063)		−0.297^***^ (0.063)
Ethnicity		0.168 (0.127)		0.103 (0.127)
Education		−0.156^***^ (0.020)		−0.164^***^ (0.02)
ln_pension		0.009 (0.008)		0.011 (0.008)
ADL		0.400^***^ (0.095)		0.451^***^ (0.096)
Hospitalization		0.377^***^ (0.054)		0.391^***^ (0.055)
Living situation		−0.116^**^ (0.054)		−0.130^**^ (0.054)
Offspring		0.018 (0.021)		0.024 (0.021)
*N*	5,706	5,706	5,706	5,706
Adj-X^2^	0.006	0.018	0.004	0.018

^*^p < 0.10, ^**^p < 0.05, ^***^p < 0.01.

### 3.2 Robustness check

#### 3.2.1 Propensity score estimation

The depressive symptoms in older people might be influenced by several circumstances, perhaps resulting in endogenous problems due to bias in sample selection. To address potential endogeneity issues and guarantee the robustness of the baseline regression results, this study includes a wide range of covariates within the model and utilizes five distinct matching methods: k-nearest neighbor matching (*k* = 1), k-nearest neighbor matching (*k* = 3), kernel matching, radius matching (cal = 0.01), and local linear regression matching. Table 3 reports the results of the propensity score estimation.

**Table 3:** Propensity score (adopted home modifications vs. non-home modifications).

Variable	Coef.	Std. Err.	*p*-value	95% CI
Age	0.035	0.005	0.000	0.025, 0.045
Gender	−0.202	0.062	0.001	−0.323, −0.080
Married	−0.010	0.081	0.905	−0.169, 0.149
Ethnicity	0.576	0.161	0.000	0.262, 0.891
Education	0.303	0.026	0.000	0.252, 0.354
ln_pension	0.090	0.010	0.000	0.000, 0.070
ADL	0.889	0.139	0.000	0.618, 1.160
Hospitalization	0.050	0.070	0.473	−0.087, 0.188
Living situation	0.260	0.070	0.000	0.124, 0.397
Offspring	−0.230	0.026	0.000	−0.281, −0.179

#### 3.2.2 Balance check

**Figure 2:**
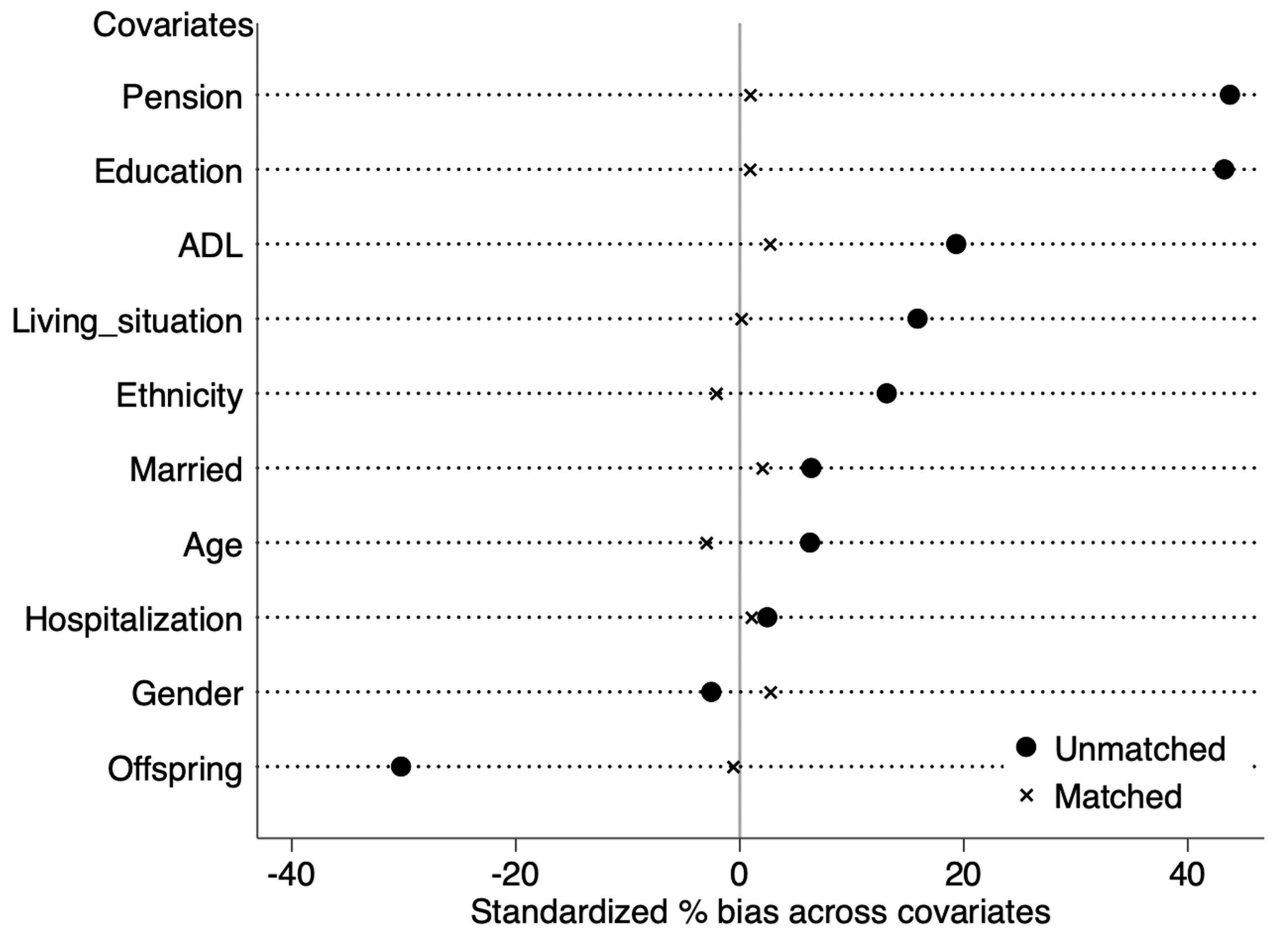
Propensity distributions of treated and control groups before and after matching.

To mitigate the potential bias in the selection of older people who implemented home modification, the PSM method was used to calculate the average impact of the change on depression among older persons. The equilibrium test outcomes of the specimens are presented in Table 4. Table 4 demonstrates a notable decrease in the standard deviation of the variables following the matching process. As illustrated in Table 4 and Figure 2, all variables standardized mean differences (%bias) were below 5% following the matching procedure. The study found no significant differences between the treatment and control groups, which confirms that the assumption of parallelism is met and suggests that there is a balance in the covariates.

**Table 4:** Parallel hypothesis in estimating the ATT of HM on the depression.

Variable	Unmatched matched	Mean		Bias%	Reduced	*T*-value	*p*-value
		Treated	Control		Bias%		
Age	U	71.332	70.874	6.3		2.19	0.028
	M	71.322	71.541	−3.0	52.2	−1.29	0.196
Gender	U	0.476	0.488	−2.5		−0.90	0.370
	M	0.475	0.469	1.2	53	0.53	0.599
Married	U	0.725	0.696	6.4		2.26	0.024
	M	0.725	0.716	2.0	68.8	0.89	0.024
Education	U	3.412	2.885	43.3		15.30	0.000
	M	3.411	3.400	0.9	97.9	0.40	0.690
Ethnicity	U	0.976	0.952	13.1		4.92	0.000
	M	0.976	0.980	−2.1	83.9	−1.18	0.239
Offspring	U	2.137	2.523	−30.2		−10.77	0.000
	M	2.138	2.145	−0.6	98	−0.28	0.778
Pension	U	7015.5	4863.5	43.8		15.53	0.000
	M	7010.5	6965.7	0.9	97.9	0.41	0.685
ADL	U	0.091	0.043	19.3		6.44	0.000
	M	0.090	0.084	2.7	86.2	1.04	0.299
Living situation	U	0.556	0.477	15.9		5.60	0.000
	M	0.556	0.556	0.1	99.3	0.05	0.959
Hospitalization	U	0.274	0.263	2.5		0.86	0.388
	M	0.274	0.269	1.0	58.2	0.45	0.653

##### 3.2.3 Estimation of treatment effects

**Figure 3:**
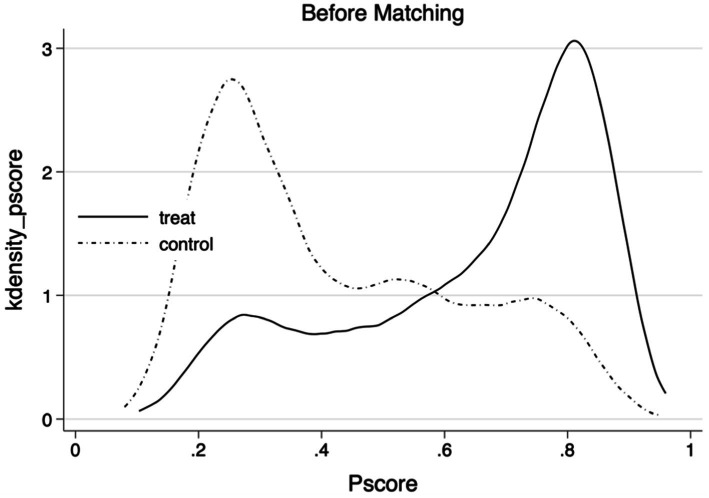
Plot of kernel density function before matching.

**Figure 4:**
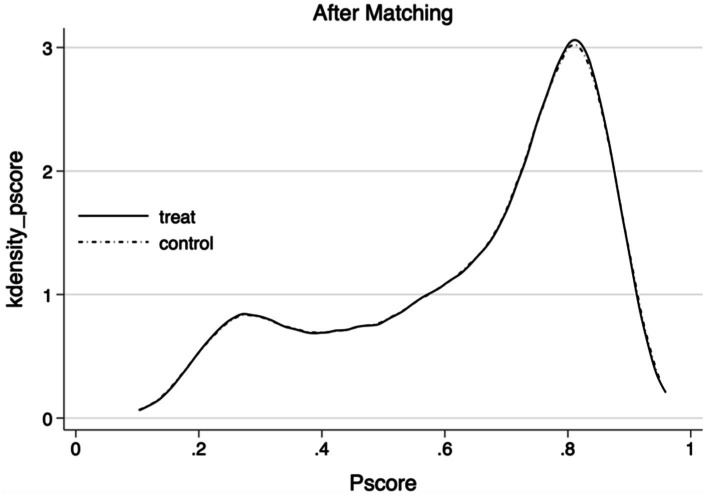
Plot of kernel density function after matching.

The Table 5 presents the average treatment effect on the treated (ATT) for home modifications, calculated using five distinct matching methods. These methods include K-nearest neighbor matching (*K* = 1), K-nearest neighbor matching (*K* = 3), kernel function matching, radius matching, and local linear regression matching. The ATT estimates are −1.168, −1.116, −1.081, −1.083, and −1.138, respectively. Each ATT estimate is statistically significant at the 0.01 level, demonstrating a notable reduction in depressive symptoms associated with home modifications. These findings reinforce the baseline regression results and corroborate the conclusions of this study. Figures 3, 4 depict the density distribution of propensity scores before and after kernel matching, highlighting enhanced balance and matching effectiveness when compared to the distribution before matching.

**Table 5:** Results of propensity score matching estimations.

Matching method	Treatment group	Control group	ATT	SE	*T* value
K-nearest neighbor matching (*K* = 1)	15.017	16.185	−1.168***	0.129	−9.050
K-nearest neighbor matching (*K* = 3)	15.017	16.132	−1.116***	0.112	−9.930
Kernel matching	15.017	16.098	−1.081***	0.099	−10.93
Radius matching	15.017	16.099	−1.083***	0.102	−10.65
Local linear regression matching	15.017	16.154	−1.138***	0.129	−8.820

*p < 0.10, **p < 0.05, ***p < 0.01; standard error after matching is obtained by bootstrap method.

## 4 Discussion

This study performed an empirical analysis to investigate the association between home modifications and the mental health of older people in China, utilizing the 2018 CLASS database and applying PSM to control for potential confounding variables. The results indicate that home modifications are significantly associated with lower depression levels in older people living in urban areas of China. Specifically, those residing in homes with a greater extent of modifications reported experiencing lower levels of depression. These findings suggest that enhancing the living environment through targeted adaptations is associated with improved mental health and well-being among older people.

The findings of this study have both practical and theoretical implications. Theoretically, the study supports the Environmental Press Theory, positing that a well-adapted environment can enhance an individual’s competence and reduce environmental stressors, leading to better psychological outcomes (52). The significance of these findings is highlighted by the background of a swiftly aging worldwide population. For example, the number of older people in the United States is expected to increase by over two-fold, rising from 40.3 million in 2010 to 85.7 million by 2050. Concurrently, the proportion of the overall population is projected to rise from 13 to 22% (51). This demographic shift underscores the increasing need for homes that accommodate the specific needs of older people.

Based on the theoretical insights, the study offers practical implications for policymakers and practitioners. Home modifications may enhance the safety and convenience of the living environment, thereby lowering the occurrence and frequency of falls and other mishaps, which are prevalent concerns for older people. The reduction of risk can alleviate older people’ anxiety and stress, contributing to lower levels of depression. To advance this, the government can integrate age-friendly modifications into public health policies, combining them with mental health services to create a comprehensive support system for older people. In terms of funding, targeted financial measures, such as subsidies, tax incentives, or low-interest loans, should prioritize vulnerable groups, including the oldest-old, functionally impaired, and disabled older people. These measures could reduce the financial burden on older households, enabling wider adoption of age-friendly modifications. Beyond financial and technical support, the government needs to establish community-based mental health services, including counseling centers, that can help older people navigate the psychological stress of environmental changes. Establishing mental health guidance and counseling centers for seniors within communities could provide ongoing psychological support, helping balance between physical and mental well-being.

In cities where the older people is growing rapidly, the government should incorporate age-friendly standards into urban planning and construction, ensuring that new housing projects and the renovation of old neighborhoods fully address the specific needs of older people. For instance, armrests at dining table edges can be integrated to assist with standing up, and bedside armrests and motion-sensor nightlights can be added in bedrooms to accommodate vision impairments. The design of age-friendly facilities should not only meet the physical needs of the older people but also their psychological needs. Additionally, raising awareness among family members about the importance of home modifications is crucial. Since many older people depend on family caregivers or live with their children, policies should recognize and strengthen the role of family members in the modification process. Training on modification techniques and offering financial support to caregivers can help them create better living environments for older people and benefit both older people’ and caregivers’ mental health.

The results of this investigation align with prior studies conducted internationally, showing a positive relationship between home modifications and reduced depression among older people. For instance, similar findings have been reported in Western countries, where aging-accessible modifications like wider hallways, handrails in showers, and first-floor bedrooms have enhanced health outcomes and comfort for older people (53, 54). However, the cultural and social context in China, including the emphasis on family support and the relatively recent focus on home modifications, may influence the extent and nature of these effects. This study enhances the existing body of knowledge by presenting empirical data from a Chinese context, highlighting the universal benefits of creating older-friendly environments.

This study has several limitations. First, the use of cross-sectional data from the CLASS database limits the ability to draw causal conclusions. While the study identifies associations between home modifications and depression in older people, it cannot fully establish directionality or underlying mechanisms. Longitudinal studies would be valuable in exploring the long-term effects and causal relationships of these modifications on mental health. Second, the CLASS data does not include pre-modification home conditions, meaning that this study compares older people living in homes with age-friendly modifications to those in homes without such features. However, it does not capture dynamic changes in home environments over time, which restricts the ability to assess the impact of the modification process itself on mental health. Despite this, the study provides important insights into the association between home modifications and depression. Future research utilizing longitudinal data could address these gaps and offer a more comprehensive understanding of the causal effects of home modifications.

These issues can be partially attributed to the limited availability of data and the absence of suitable instrumental factors regarding occupational stratification in Chinese social surveys. Furthermore, additional investigation is required to delve into the study of the mechanism. This study examines the association between HM and depression levels in older people. Nevertheless, intriguing mediating mechanisms have not been well investigated, such as the quantity of familial support received and the individual’s ability to search for information. These constraints underscore the need for additional investigation and should be tackled in forthcoming research endeavors.

In conclusion, the study provides evidence of a significant association between home modifications and reduced depression levels in older people in China. The result emphasizes the necessity of creating supportive living environments to enhance mental health and well-being among the aging population. With the global issue of aging populations, bridging the gap between current services and the growing requirements of older people is crucial. The benefit of HM on mental health requires policy to pay attention to the improvements in physical environments and a comprehensive approach that includes psychological support and the promotion of digital literacy among older people.

## 5 Conclusion

This study investigates the impact of home modifications on depression levels of older people in China. Using the 2018 China Longitudinal Aging Social Survey database, the findings indicate that home modifications are significantly associated with reduced depression levels among older people, highlighting their positive role in improving mental health of older people. Moreover, there is an inverse relationship between the degree of home modifications and the levels of depression in older people. The conclusion remains valid after conducting a robustness examination using propensity score matching. The study underscores the crucial positive effects of home modifications on older people’s mental health, offering a theoretical foundation for the future promotion and implementation of these modifications. By emphasizing the value of targeted interventions, this study provides actionable insights for policymakers and practitioners aiming to foster an age-friendly environment that supports successfully aging in place.

## Data Availability

Publicly available datasets were analyzed in this study. This data can be found here: http://class.ruc.edu.cn.

## References

[1] Zrinščak S Lawrence S. Active ageing and demographic change: challenges for social work and social policy. Eur J Soc Work. (2014) 17:313–21. doi: 10.1080/13691457.2014.919088

[2] Ratnayake M Lukas S Brathwaite S Neave J Henry H. Aging in place: are we prepared? Delaware J Public Health. (2022) 8:28–31. doi: 10.32481/djph.2022.08.007, PMID: 36177171 PMC9495472

[3] Ahn M Kwon HJ Kang J. Supporting aging-in-place well: findings from a cluster analysis of the reasons for aging-in-place and perceptions of well-being. J Appl Gerontol. (2020) 39:3–15. doi: 10.1177/0733464817748779, PMID: 29277156

[4] United Nations. Political declaration and Madrid international plan of action on ageing. (2011). Available online at: https://www.un.org/esa/socdev/documents/ageing/MIPAA/political-declaration-en.pdf (Accessed April 23, 2025).

[5] Bonnefoy X. Inadequate housing and health: an overview. Int J Environ Pollut. (2007) 30:411–29. doi: 10.1504/IJEP.2007.014819, PMID: 35009967

[6] Arku G Luginaah I Mkandawire P Baiden P Asiedu AB. Housing and health in three contrasting neighbourhoods in Accra, Ghana. Soc Sci Med. (2011) 72:1864–72. doi: 10.1016/j.socscimed.2011.03.023, PMID: 21561698

[7] Vespa J Engelberg J He W. Old housing, new needs: are US homes ready for an aging population. Curr Pop Rep. (2020):23–217.

[8] Lee S Oh E Hong GR. Comparison of factors associated with fear of falling between older adults with and without a fall history. Int J Environ Res Public Health. (2018) 15:982. doi: 10.3390/ijerph15050982, PMID: 29757960 PMC5982021

[9] Ryu E Juhn YJ Wheeler PH Hathcock MA Wi CI Olson JE. Individual housing-based socioeconomic status predicts risk of accidental falls among adults. Ann Epidemiol. (2017) 27:415–420.e2. doi: 10.1016/j.annepidem.2017.05.019, PMID: 28648550

[10] Hyun KR Kang S Lee S. Population aging and healthcare expenditure in Korea. Health Econ. (2015) 25:1239–51. doi: 10.1002/hec.3209, PMID: 26085120

[11] Wang L Chen J Ju DY. Factors contributing to Korean older adults’ acceptance of assistive social robots. Electronics. (2021) 10:2204. doi: 10.3390/electronics10182204

[12] Xinhuanet. 40 million older adults fall every year. Why? (2020). Available online at: http://www.xinhuanet.com/politics/2020-09/07/c_1126461988.htm (In Chinese; accessed April 23, 2025).

[13] Gerards MH McCrum C Mansfield A Meijer K. Perturbation-based balance training for falls reduction among older adults: current evidence and implications for clinical practice. Geriatr Gerontol Int. (2017) 17:2294–303. doi: 10.1111/ggi.13082, PMID: 28621015 PMC5763315

[14] Drozdick LW Edelstein BA. Correlates of fear of falling in older adults who have experienced a fall. J Clin Geropsychol. (2001) 7:1–13. doi: 10.1023/A:1026487916681

[15] Hoang OT Jullamate P Piphatvanitcha N Rosenberg E. Factors related to fear of falling among community-dwelling older adults. J Clin Nurs. (2016) 26:68–76. doi: 10.1111/jocn.13337, PMID: 27723217

[16] Deshpande N Metter EJ Bandinelli S Lauretani F Windham BG Ferrucci L. Psychological, physical, and sensory correlates of fear of falling and consequent activity restriction in the elderly. Am J Phys Med Rehabil. (2008) 87:354–62. doi: 10.1097/PHM.0b013e31815e6e9b, PMID: 18174852 PMC2495025

[17] Scheffer AC Schuurmans MJ van Dijk N van der Hooft T de Rooij SE. Fear of falling: measurement strategy, prevalence, risk factors and consequences among older persons. Age Ageing. (2008) 37:19–24. doi: 10.1093/ageing/afm169, PMID: 18194967

[18] Sugiyama T Thompson CW. Outdoor environments, activity and the well-being of older people: conceptualising environmental support. Environment and Planning A (2007) 39: 1943–1960. doi: 10.1068/a38226

[19] AOTA. Home modifications and occupational therapy. (2016). Available online at: https://www.aota.org/about-occupational-therapy/professionals/rdp/homemods.aspx (Accessed April 14, 2020).

[20] Hwang E Cummings L Sixsmith A Sixsmith J. Impacts of home modifications on aging-in-place. J Hous Elder. (2011) 25:246–57. doi: 10.1080/02763893.2011.595611

[21] World Health Organization. Global age-friendly cities: a guide. Geneva: WHO (2007).

[22] Rubinstein RL. The home environments of older people: a description of the psychological process linking person to place. J Gerontol. (1989) 44:S45–53. doi: 10.1093/geronj/44.2.S45, PMID: 2921478

[23] Lawton MP. Residential environment and self-directedness among older people. Am Psychol. (1990) 45:638–40. doi: 10.1037/0003-066X.45.5.638, PMID: 2350081

[24] Gitlin LN. Conducting research on home environments: lessons learned and new directions. The Gerontologist. (2003) 43:628–37. doi: 10.1093/geront/43.5.628, PMID: 14570959

[25] Oswald F Schilling O Wahl HW Fänge A Sixsmith J Iwarsson S. Homeward bound: introducing a four-domain model of perceived housing in very old age. J Environ Psychol. (2006) 26:187–201. doi: 10.1016/j.jenvp.2006.07.002

[26] Oswald F Wahl HW Schilling O Iwarsson S. Housing-related control beliefs and independence in activities of daily living in very old age. Scand J Occup Ther. (2007) 14:33–43. doi: 10.1080/11038120601151615, PMID: 17366076

[27] Oswald F Wahl HW Schilling O Nygren C Fänge A Sixsmith A. Relationships between housing and healthy aging in very old age. Gerontologist. (2007) 47:96–107. doi: 10.1093/geront/47.1.96, PMID: 17327545

[28] Oswald F Wahl HW. Dimensions of the meaning of home in later life In: Rowles GD Chaudhury H, editors. Home and identity in later life: international perspectives. New York, NY: Springer (2005). 21–46.

[29] Wiles JL Leibing A Guberman N Reeve J Allen RE. The meaning of ‘aging in place’ to older people. The Gerontologist. (2012) 52:357–66. doi: 10.1093/geront/gnr098, PMID: 21983126

[30] Stones D Gullifer J. ‘At home it’s just so much easier to be yourself’: older adults’ perceptions of ageing in place. Ageing Soc. (2016) 36:449–81. doi: 10.1017/S0144686X14001214

[31] Vaishya R Vaish A. Falls in older adults are serious. Indian J Orthop. (2020) 54:69–74. doi: 10.1007/s43465-019-00037-x, PMID: 32257019 PMC7093636

[32] Ellison C Struckmeyer L Kazem-Zadeh M Campbell N Ahrentzen S Classen S. A social-ecological approach to identify facilitators and barriers of home modifications. Int J Environ Res Public Health. (2021) 18:8720. doi: 10.3390/ijerph18168720, PMID: 34444467 PMC8391256

[33] Kim D Portillo M. Fall hazards within senior independent living: a case-control study. Health Environ Res Design J. (2018) 11:65–81. doi: 10.1177/1937586717754185, PMID: 29417846

[34] McCullagh MC. Home modification: how to help patients make their homes safer and more accessible as their abilities change. Am J Nurs. (2006) 106:54–63. doi: 10.1097/00000446-200610000-00033, PMID: 17016095

[35] Kruse RL Moore CM Tofle RB LeMaster JW Aud M Hicks LL. Older adults’ attitudes toward home modifications for fall prevention. J Hous Elder. (2010) 24:110–29. doi: 10.1080/02763891003757031

[36] Kim H Ahn YH Steinhoff A Lee KH. Home modification by older adults and their informal caregivers. Arch Gerontol Geriatr. (2014) 59:648–656. doi: 10.1016/j.archger.2014.07.012 25109810

[37] Johansson K Borell L Lilja M. Older persons’ navigation through the service system towards home modification resources. Scand J Occup Ther. (2009) 16:227–37. doi: 10.3109/11038120802684307 19148848

[38] Chen QH Lin H Lv ZW. Does village democracy increase happiness? Evidence from rural China. China Econ Q. (2014) 13:723–44. doi: 10.13821/j.cnki.ceq.2014.02.005

[39] Radloff LS. The CES-D scale: a self-report depression scale for research in the general. Appl Psych Meas. (1977) 1:385–401. doi: 10.1177/014662167700100306

[40] Thapa DK Visentin DC Kornhaber R Cleary M. Prevalence and factors associated with depression, anxiety, and stress symptoms among older adults: a cross-sectional population-based study. Nurs Health Sci. (2020) 22:1139–52. doi: 10.1111/nhs.12783, PMID: 33026688

[41] Sagna A Gallo JJ Pontone GM. Systematic review of factors associated with depression and anxiety disorders among older adults with Parkinson’s disease. Parkinsonism Relat Disord. (2014) 20:708–15. doi: 10.1016/j.parkreldis.2014.03.020 24780824 PMC4648277

[42] Osborn DPJ Fletcher AE Smeeth L Stirling S Bulpitt CJ Breeze E. Factors associated with depression in a representative sample of 14,217 people aged 75 and over in the United Kingdom: results from the MRC trial of assessment and management of older people in the community. Int J Geriatr Psychiatry. (2003) 18:623–30. doi: 10.1002/gps.896, PMID: 12833307

[43] Yang L Zikos V. Healthy mind in healthy body: identifying the causal effect of mental health on physical health. Econ Lett. (2022) 213:110358. doi: 10.1016/j.econlet.2022.110358

[44] Chen X Wang T Busch SH. Does money relieve depression? Evidence from social pension expansions in China. Soc Sci Med. (2023) 317:115516. doi: 10.1016/j.socscimed.2018.12.004 PMC6800113 30530234

[45] Chen X Wang TY Busch SH. Psychosocial well-being associated with activity of daily living stages among community-dwelling older adults. Gerontol Geriatr Med. (2017) 3:2333721417700011. doi: 10.1177/2333721417700011, PMID: 28540343 PMC5433668

[46] Meuleners LB Fraser ML. Gender differences in recurrent mental health contact after a hospitalization for interpersonal violence: Western Australia, 1997 to 2008. J Interpers Violence. (2015) 30:333–47. doi: 10.1177/0886260514534779, PMID: 24870962

[47] Barrett AE Turner RJ. Family structure and mental health: the mediating effects of socioeconomic status, family process, and social stress. J Health Soc Behav. (2005) 46:156–69. doi: 10.1177/002214650504600203, PMID: 16028455

[48] Steptoe A Shankar A Demakakos P Wardle J. Social isolation, loneliness, and all-cause mortality in older men and women. Proc Natl Acad Sci USA. (2013) 110:5797–801. doi: 10.1073/pnas.1219686110, PMID: 23530191 PMC3625264

[49] Evenson RJ Simon R. Clarifying the relationship between parenthood and depression. J Health Soc Behav. (2005) 46:341–58. doi: 10.1177/002214650504600403, PMID: 16433280

[50] Ross C Mirowsky J Goldsteen K. The impact of the family on health: the decade in review. J Marriage Fam. (1990) 52:1059–78. doi: 10.2307/353319

[51] Umberson D Gove W. Parenthood and psychological well-being: theory, measurement, and stage in the family life course. J Fam Issues. (1989) 10:440–62. doi: 10.1177/019251389010004002

[52] Rosenbaum PR Rubin DB. The central role of the propensity score in observational studies for causal effects. Biometrika. (1983) 70:41–55. doi: 10.1093/biomet/70.1.41

[53] Miller WC Anton HA Townson A. Measurement properties of the CES-D scale among individuals with spinal cord injury. Spinal Cord. (2008) 46:287–92. doi: 10.1038/sj.sc.3102127, PMID: 17909558

[54] Wolin KY Yan Y Colditz GA Lee IM. Physical activity and colon cancer prevention: a meta-analysis. Br J Cancer. (2009) 100:611–6. doi: 10.1038/sj.bjc.6604917, PMID: 19209175 PMC2653744

[55] Cohen S Evans GW Stokols D Krantz DS. Behavior, health, and environmental stress. New York: Plenum Press (1986).

